# Optimization of BeWo model to investigate placental responses to *Plasmodium falciparum* infected erythrocytes

**DOI:** 10.5281/zenodo.10757455

**Published:** 2017-03-18

**Authors:** Winifrida Kidima, Naveen Bobbili, Diane W. Taylor

**Affiliations:** 1 Department of Tropical Medicine Medical Microbiology and Pharmacology, University of Hawai’i at Manoa, 651 Ilalo Street Honolulu, Hawaii 98213 USA; 2 University of Dar es Salaam, College of Natural and Applied Sciences, Department of Zoology and Wildlife Conservation, P.O. Box 35064, Tanzania

## Abstract

**Background:**

Establishment of an *in vitro* model to study placental malaria is essential for understanding the biology and pathogenesis of placental malaria. We defined experimental variables for obtaining responses of BeWo cells to placental binding *Plasmodium falciparum* infected erythrocytes (IE, CS2 parasites).

**Materials and methods:**

Experimental variables included i) concentration of forskolin, a c*yclic adenosine monophosphate inducer* important in the induction of syncytialisation of BeWo, ii) suitable period of incubating BeWo with forskolin, and iii) ratio of IE to BeWo cells and length of incubation to induce physiological changes in BeWo cells, including the vasculogenic factors vascular endothelial growth factor A (VEGFA), endoglin, and angiopoietin-2; an anti-angiogenic factor (inhibin A); a regulator of cell growth, mammalian target of rapamycin (mTOR); a chemokine (IL-8); and the cytokine macrophage inhibition factor. The human hormone, chorionic gonadotrophin was used as a marker for syncytialisation.

**Results:**

We showed that 72 hrs incubation of BeWo with 10 μm forskolin resulted in higher levels of syncytialisation and hCG secretion. Overall, the best condition was to co-culture syncytialised BeWo with a 10:1 ratio of IE for 48 hours. Under these conditions, when co-cultured with IE, BeWo produced increased amounts of IL-8 (p=0.0001), VEGF (p=0.001) and endoglin (p=0.001).

**Conclusion:**

The model can be used to evaluate the impact of IE, inflammatory cytokines and other factors associated with placental malaria on syncytiotrophoblast function.

## 1 Introduction

Pathogenesis of placental malaria may occur when *P. falciparum* infected erythrocytes (IE) bind to syncytiotropho-blasts (ST), which results in sequestration of IE in the intervillous space. As a result, infiltration of maternal monocytes may occur that secrete proinflammatory cytokines causing pathology. The binding of IE to ST is mediated by the parasite protein VAR2CSA, which is expressed on the surface of IE, with a receptor called chondroitin sulphate A (CSA) on ST [1-4]. Therefore, during placental malaria (PM), fetal ST interact with IE, maternal monocytes, and inflammatory cytokines secreted by maternal monocytes and ST. In malaria endemic areas, the major outcome of the interaction is low birth weight babies [5] as a result of preterm delivery and fetal growth restriction. Little is known about the pathogenesis of placental malaria that leads to low birth weight babies, particularly the patho-physiological changes in ST that result from their interaction with *P. falciparum* IE.

One approach to investigate the pathogenesis of PM would be *in vivo* studies using an animal model. However, the human ST are, in many aspects, different from rodent ST and rodents are a poor model for studying human placental malaria [6]. In addition, the variable surface antigens (VSA) that mediate binding to ST in murine malaria leading to sequestration in the intervillous space differ between human and rodent malaria parasites [7]. While the human perfusion model and trophoblasts primary cells are an alternative for *ex vivo* and *in vitro* studies of placental malaria pathogenesis, human ST from term placentas have a very short life span in culture and are in a senescent state at delivery. BeWo cells are a stable cytotrophoblast cell line capable of acquiring the ST phenotypic and endocrine characteristics upon treatment with forskolin, an inducer of cyclic adenosine monophosphate (cAMP). The BeWo system has recently been used as an *in vitro* model to select *P. falciparum* placental binding parasites [8] as well as in studies on placental malaria pathogenesis [9-11]. However, there are no published data concerning optimisation of BeWo cell lines to study placental malaria pathogenesis. The optimisation of the BeWo model and development of associated *in vitro* protocols will allow for the study of pathophysiological changes in ST associated with placental malaria that lead to poor pregnancy outcomes.

In this study, we sought to optimise various experimental variables and establish an *in vitro* system that mimics different aspects of the malaria induced microenvironment in which malaria parasites mediate placental pathology. Gene expression and protein assays were used to study the dynamics of BeWo responses to various concentrations of intact IE at different time points. Several genes whose proteins are reported to be dysregulated in pregnant women during placental malaria [12-14] and factors important for placental and fetal growth were studied. The genes included: vascular endothelia growth factor (VEGF) A, which is important in inducing angiogenesis, vasculogenesis and mediates vascular permeability; endoglin, a transforming growth factor co-receptor also involved in regulation of angiogenesis; angiopoietin (ANGPT)-2, a key regulator of angiogenesis; inhibin A (INHA), an anti-angiogenic molecule; and mammalian target of rapamycin (mTOR) that is implicated in the regulation of growth *in utero.* In addition, IL-8 and macrophage inhibitory factor (MIF), which are elevated during placental malaria [13,15] were measured. Overall, the study sought to determine the optimal number of intact IE required to induce responses to BeWo cells and the length of time required for the changes to occur.

## 2 Material and methods

### 2.1 Parasite culture and BeWo cell line

The CS2 strain of *P. falciparum* was grown in type O+ blood at 5% haematocrit in RPMI-1640 media (Invitrogen) supplemented with 50 μg/ml hypoxanthine (Sigma), 0.1 mg/ml gentamicin sulphate (Sigma), 0.5% albumax, and 5% human AB serum at 37oC in in the presence of 5% CO_2_, 90% N2 and 5% O_2_.

BeWo cells, obtained from ATCC, were grown in Ham’s F12 medium supplemented with 10% heat-inactivated fetal bovine serum, 1% L-glutamine and 1% penicillin/streptomycin (Invitrogen), at 37°C in the presence of 5% CO_2_. For all experiments, BeWo were seeded in 24-well plates at 2.0 x 10^5^ cells/well and incubated until the cells were 90% confluent, before being treated with 10 μm forskolin. In parallel experiments, cell viability for the different treatments was assessed using 96 well plates, initially seeded with 1 x 10^4^ cells/well.

### 2.2 Optimal concentration of forskolin and incubation time with BeWo cells

To determine the effective concentration and timing of forskolin treatment required for BeWo to acquire pheno-typic and endocrine characteristics of ST, the concentration of β-human chorionic gonadotrophin hormone (hCG) secreted by the cells into culture supernatants was measured using a commercial ELISA kit (Alpha diagnostic). BeWo cells were treated with different concentrations of forskolin, including 10, 25 or 50 μm forskolin (Calbiochem) and 25 μm DMSO (the vehicle for for-skolin) in Hams F12 medium. Forskolin treated BeWo cells were incubated for 24, 48, 72 or 96 hrs in triplicate and then pooled for analysis. Viability of BeWo cells was determined using methylthiazolyldiphenyl-tetrazolium bromide (MTT) assay [16-18].

### 2.3 Magnetic purification technique

To concentrate trophozoite stage IE that express VAR2CSA that binds to CSA on ST, CS2 parasites were cultured *in vitro* and purified using a magnetic column (LS Columns Miltenyi Biotec) with Quadro MACS separator. The columns positively purify trophozoite stage parasites. CS2 IE from synchronised cultures were washed with PBS. The IE pellet was then suspended in sterile filtered MACS buffer (PBS, pH 7.2, 0.5% BSA, 2 Mm EDTA) and MACS buffer was added dropwise to pre-wet the columns. Then, the MACS parasite suspension was added to the columns attached to MACS separator, and the flow-through containing ring stage parasites was discarded. Before eluting the trophozoites, the columns were detached from the Quadro MACS separator and the trophozoite stage parasites were eluted with 5 ml MACS buffer and collected in a 15 ml tube. The eluted IE were then centrifuged at 2500 rpm for 5 min, supernatant removed and parasitemia was determined using microscopy. The eluate consisted of trophozoites, schizonts, free hemozoin and parasite debris (hemozoin DNA complexes, parasite DNA).

### 2.4 Timing of BeWo responses toward intact infected erythrocytes

To determine the time point at which IE induce detectable changes in forskolin treated BeWo cells, three different concentrations of intact IE and four different incubation times were utilised. Briefly, BeWo cells were seeded in 24 well plates at 2 x 10^5^ cells/well and treated with forskolin when the cells reached 90% confluence, for 72 hrs at 37ºC. Then, the forskolin treated BeWo were incubated with intact IE at ratios of 10, 1, and 0.1 IE per 1 BeWo for 6, 12, 24 or 48 hrs. Normal cultured red blood cells (nRBC) were used as controls. Both protein assay (ELISA and Luminex) and gene expression (RT-PCR) were used to measure expression of selected markers; i.e., measured gene transcription and amount of protein secreted.

### 2.5 ELISA assay for MIF detection in supernatant

The amount of MIF, a cytokine that is elevated in malaria positive placentas, was measured in the supernatant using an ELISA kit (R&D, Duoset Cat No DY289). Briefly, 2 μg/μl of mouse anti human MIF capture antibody was used for coating ELISA plates (Maxisorp) and incubated at 4˚C overnight. The capture antibody was then aspirated and the wells washed three times with 0.05% Tween in PBS. Then, 300 μl of blocking solution 1% BSA in PBS (filtered with 0.2 μm) was added and incubated for 2 hrs at 37ºC. The blocking solution was aspirated and wells washed three times with 0.05% Tween in PBS. Samples (pooled supernatants from triplicate wells) and standards were then added and incubated for 2 hrs at 37ºC. (This experiment was regarded as descriptive so only single assays of pooled samples were measured). After aspirating and washing, 100 μl of 100 ng/ml of biotinylated goat anti human MIF detection antibody was added and incubated for 2 hrs. Then, 100μl of streptavidin-HRP at a 1:200 dilution (in diluent) was added to each well and incubated for 30 min. The intensity of the colour change was then recorded at 450/630 nm.

### 2.6 BeWo RNA extraction

Total RNA was isolated using the RNeasy kit (Qiagen) according to the manufacturer’s protocol. Quality and yield of RNA was assessed using a nanodrop UV spectrophotometer. RNA preparations were considered pure when the A260/A280 absorbance ratio was equal to 1.9-2.0. Assessment of gene expression on selected genes from stimulated forskolin treated BeWo was determined using real time PCR (RT-PCR).

### 2.7 cDNA synthesis and RT-PCR for selected markers

High quality RNA (A260/A280 ratio = 1.9-2.0) was used for cDNA synthesis that was carried out according to the manufacturer’s protocol (Quanta Bioscience). cDNA was diluted at 1:5 with nuclease free water for RT-PCR assays. Three independent amplification assays were carried out in duplicate for each gene using a total reaction volume of 20 μl. The reaction contained 1.5 μl of sample cDNA, 10 μl PerfeCTa SYBR Green SuperMix (Quanta Bioscience 59), 250 nm (0.5 μl) of gene specific forward and reverse primers (Bio-Rad) in nuclease free water. A master mix containing forward and reverse primers for *beta actin* was prepared for amplification as an internal control (reference gene). The RNA was reverse transcribed using SYBR Green qPCR under cycling conditions recommended by the manufacturer. Data were acquired using iCycler (Bio Rad). Gene expression levels were normalised using an internal control, i.e., the *beta actin* gene [19]. Mean relative RNA expression levels in reference to the internal control were determined and compared among the IE and nRBC treatment groups.

### 2.8 Luminex assay for IL-8 detection in supernatant from BeWo cultures

Using microparticles (VersaMap) coupled with IL-8 specific antibodies, IL-8 levels in BeWo culture supernatants were measured according to the manufacturer instructions. Briefly, 50 μl of IL-8 pre-coated microparticles (500 mi-crospheres/50 μl) were pipetted into 96 well plates (previously pre-wet wells with washing buffer). Then, 200 μl of samples and 50 μl of standards were added into wells containing microparticles and incubated for 2 hrs at room temperature on a horizontal orbital microplate shaker set at 500 ± 50 rpm. The liquid was then removed by vacuum aspiration and three washing steps with washing buffer were performed. For detection, 50μl of (2 μg/ml) IL 8 specific biotinylated antibody was added and incubated for one hour at room temperature on a shaker. After three washing, 50 μl of streptavidin-phycoerythrin conjugate antibody (2 μg/ml) was added and incubated for 30 min.After washing, 100 μl of washing buffer was added and median fluorescent intensity (MIF) was read using a Mi-croChip M100 reader.

### 2.9 Data analysis

All data are presented as means ± SEM. Experiments were conducted twice in triplicates. Experiments involving hCG concentration and MIF were considered descriptive where triplicate samples were pooled and only a single assay was measured. Changes in gene expression in treatment sample relative to controls were analysed using comparative C(T) method [20]. The two sample t-test was used to investigate the difference between normalised C(T)s. A two way ANOVA was used to test for difference in RNA and protein expression of the markers by BeWo induced by treatments and control followed by the Bonferonni Multiple Comparison Test using GraphPad prism version 5.03. Treatments providing the largest difference between the experimental treatment and the negative controls were considered to be optimal.

## 3 Results

### 3.1 Syncytialisation and viability of BeWo cells with forskolin

**Figure 1.**
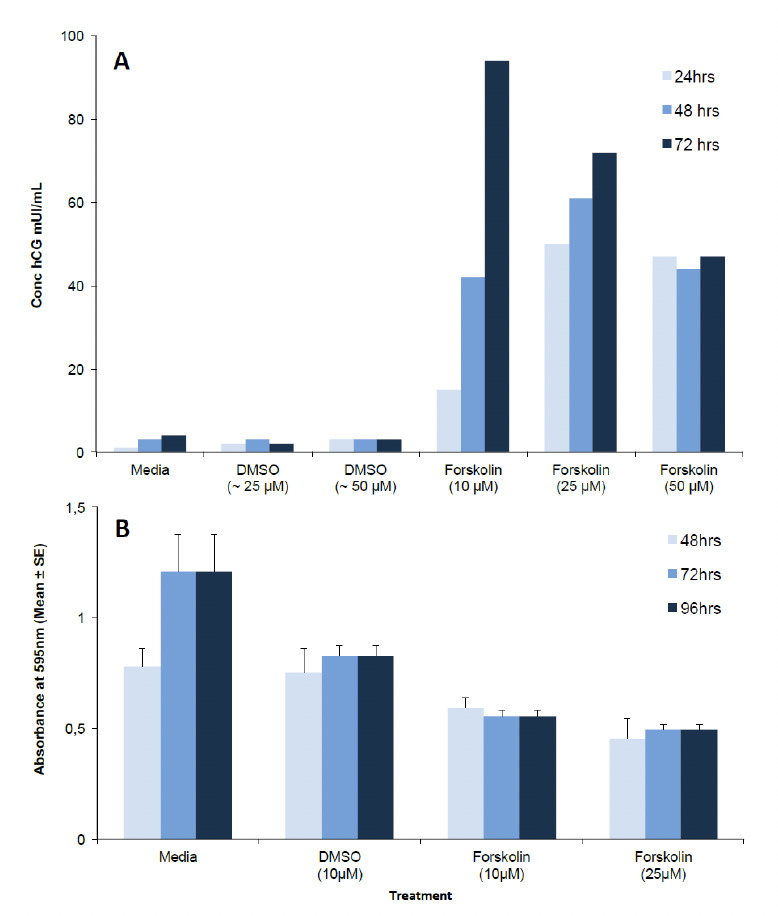
Effect of forskolin on syncytial formation of BeWo, as indicated by hCG secretion, and cell viability. A: Concentration of hCG secreted by BeWo treated with 10, 25 or 50 μm of for-skolin in DMSO. Experiment done in triplicate and pooled for analysis. B: Viability of BeWo treated with different concentrations of forskolin. 1 x 10^4^ BeWo were treated with different concentrations of forskolin in DMSO or DMSO alone for different periods of time. Data represent the mean ± SE (n=6). Two ways ANOVA-Bonferroni post test was used. No significant difference between viability of BeWo treated with 10 μm forskolin at 48, 72 and 96 hrs was observed (p=0.62). The increase in cell viability in the media (control) is due to proliferation of BeWo cells; this proliferation does not happen once syncytial formation occurs.

**Figure 2.**
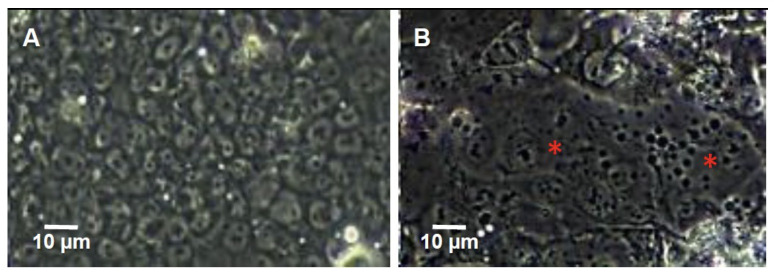
BeWo cell monolayers. A: before and, B: after treatment with 10 μm forskolin for 72 hrs. Red asterisks indicate monolayer of syncytialised BeWo.

To assess the efficiency of different concentrations of for-skolin for inducing syncytialisation of BeWo cells, levels of hCG secreted by BeWo into the supernant of BeWo that were treated with 10, 25 or 50 μm of forskolin were measured. The results showed that BeWo without forskolin secreted less hCG than BeWo treated with 10, 25 or 50 μm of forskolin (Figure 1A). In general, the amount of hCG increased with time, with higher concentrations being detected at time 72 hrs with 10 μm of forskolin. Overall, 10 μm forskolin induced 23% more hCG at 72 hrs than 25 μm. Figure 2 shows BeWo monolayer without (single cells with >95% confluence) treated with 10 μm forskolin (syncytial formation).

To investigate if cell viability was reduced with different concentrations of forskolin in DMSO, the MTT assay was used. No difference in viability of BeWo treated with different concentrations of forskolin was detected (P=0.62). Viability of BeWo treated with forskolin at 10 μm concentration was stable until 96 hrs of incubation (Figure 1B). As expected there was a clear difference in O.D. of the MTT assay between BeWo treated with for-skolin and untreated controls (p<0.01, two way ANOVA, Bonferonni multiple comparison test), because BeWo cells in the untreated controls continued to proliferate and increase in number whereas forskolin treated BeWo formed syncytia and their numbers remained constant (Figure 2). Thus, treating BeWo with 10 μm forskolin for 72 hrs was used in subsequent experiments.

### 3.2 Protein and gene expression in BeWo cells co-cultured with different numbers of intact IE for 12 and 48 hrs

**Figure 3.**
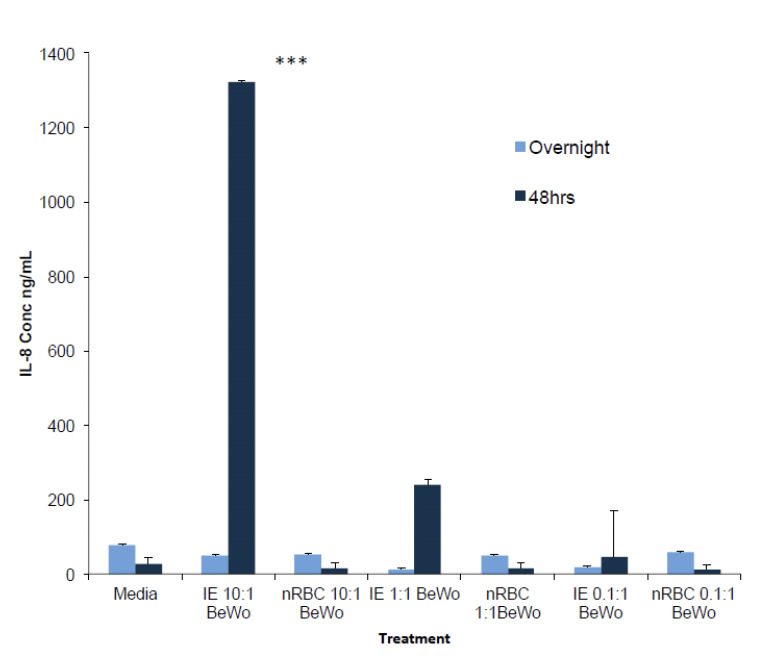
IL-8 secretions by BeWo cells treated with different concentrations of intact CS2 infected erythrocytes (IE) using normal red blood cells (nRBC) as controls. BeWo cells were treated with 10 μm forskolin, and incubated with different concentrations of IE overnight or 48 hrs. Data represent the mean ± SE (n=6). Two-way ANOVA-Bonferroni post test was used. Three asterisk indicate a significant (P<0.0001) difference between the amount of IL-8 secreted by BeWo treated with 10 IE and 10 nRBC at 48 hrs incubation.

To assess the influence the different numbers of IE on the induction of responses, forskolin treated BeWo cells were exposed to different numbers of purified IE and cultured normal RBC (controls) overnight and 48 hrs and the level of IL-8 and RNA expression of VEGFA and mTOR were measured. The results showed no change in the amount of IL-8 secreted by BeWo co-cultured overnight with 10, 1, or 0.1 intact IE (P>0.05). However, at 48 hrs, a ratio of 10 IE: 1 BeWo cells resulted in the induction of a 87-fold increase in IL-8 compared to treatment with 10 nRBC1 BeWo (Figure 3) (p<0.0001, two way ANOVA, Bonferonni multiple comparison test).

**Figure 4.**
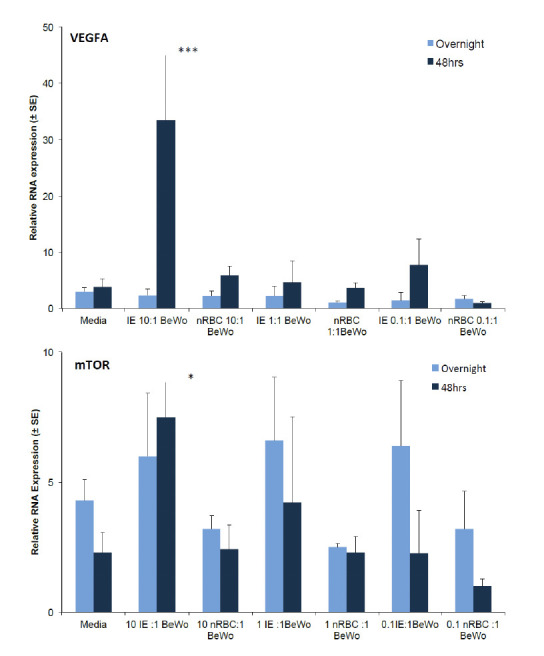
Relative RNA expression of selected markers by BeWo treated with different concentrations of intact CS2 infected erythrocytes (IE) and normal RBC (nRBC) (mean RNA expression relative to the internal control, β-actin gene). Forskolin-treated BeWo cells were cultured with different concentrations of IE and nRBC overnight or for 48 hrs. Data represent Mean ± SE (n=6). A: VEGFA, and B: mTOR (two-way ANOVA-Bonferroni post test was used. Asterisks indicate a significant difference between the mean RNA expression of VEGFA (p<0.001) and mTOR (p <0.05) by BeWo treated with 10 IE and 10 nRBC respectively, at 48 hrs post incubation.

Likewise at 48 hrs, co-culturing of forskolin treated BeWo with 10 IE resulted in a 5-fold increase in VEGFA RNA expression compared to forskolin treated BeWo treated with equivalent numbers of nRBC (10:1 IE) (p<0.001 (Figure 4). No significant differences were observed in the RNA expression of mTOR at 48 hrs, with expression being less than 2-fold between the 10:1 IE and nRBC treatment groups (P>0.05). Thus, overall, higher expression of selected genes and protein was observes at 48 hrs with 10 IE: 1 BeWo treatment compared to overnight treatment.

### 3.3 RNA expression of inhibin, ANG2, mTOR and endoglin in BeWo cells with a 10:1 IE to BeWo ratio

**Figure 5.**
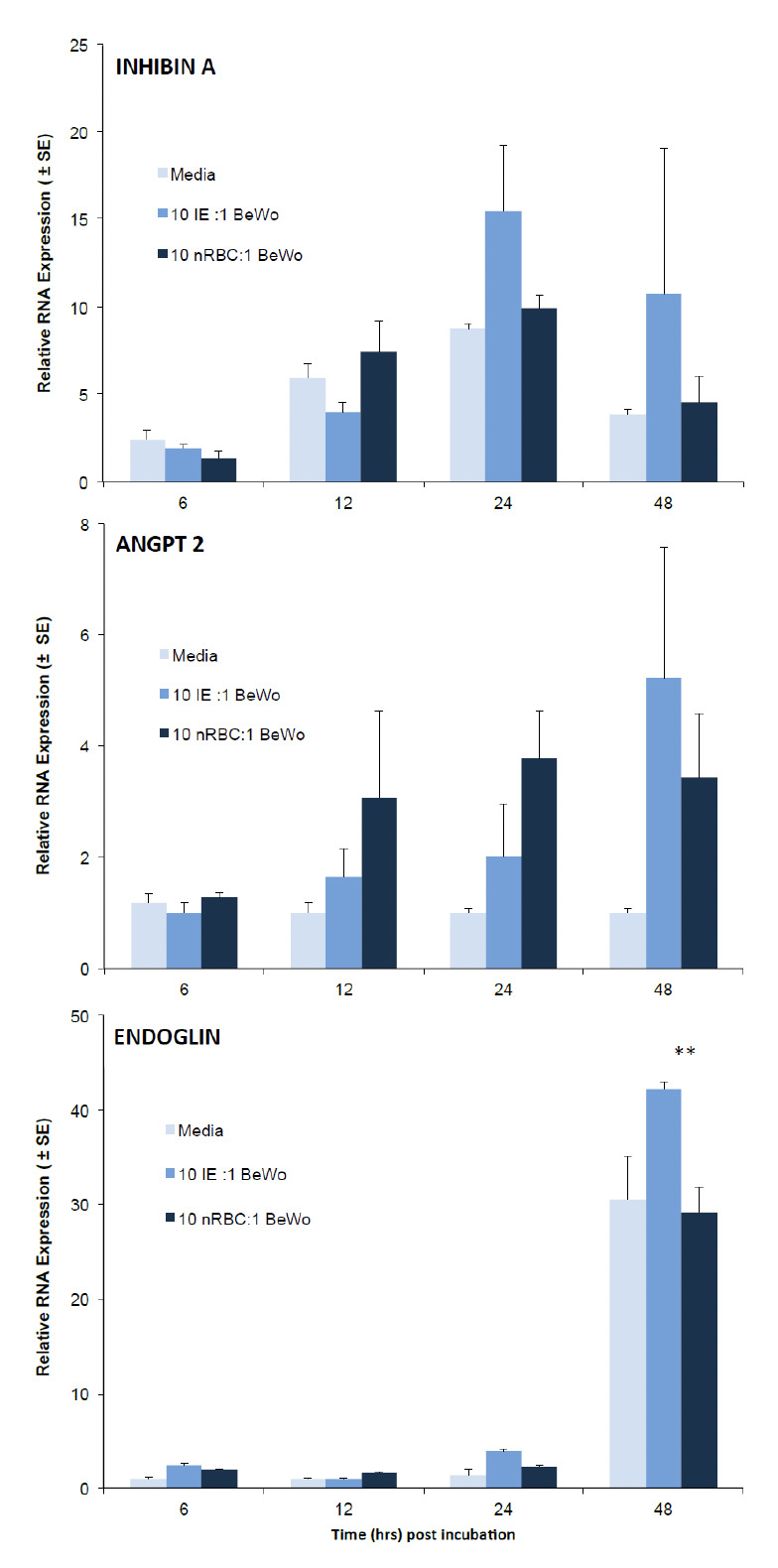
Time course expression of selected markers in BeWo cells. 2 x 10^5^ forskolin-treated BeWo were incubated with 2 x10^6^ CS2 IE using nRBC as control and incubated for 6, 12, 24 or 48 hrs. Figure shows relative levels of RNA expression of Inhibin A (top), ANGPT 2 (middle) and endoglin (bottom) relative to β-actin. Data represent mean ± SE (n=6). Two asterisks indicate a significant difference between the mean RNA expression of en-doglin (p<0.001) between BeWo treated with 10 IE and 10 nRBC respectively at 48 hrs post incubation (two-way ANOVA with Bonferroni post test).

Since 10:1 IE ratio induced significant changes in gene expression at 48 hrs, we analysed the expression of other genes (markers) in forskolin treated BeWo at different time points at a 10 IE: 1 BeWo ratio using the same number of nRBC as control treatment. RNA expression levels of endoglin in all treatment groups were higher at 48 hrs regardless of the treatment, with 10:1 IE inducing more endoglin than 10:1 nRBC (p<0.001). With IHBA, RNA expression was higher in BeWo treated with 10 IE at both 24 and 48 hrs with 10:1 IE treatment inducing at least 2-fold more RNA compared to nRBC controls but the differences were not significant (p>0.05; Figure 5).

RNA expression levels and pattern of ANGPT-2 were similar at 6 hrs. Changes in ANGPT-2 RNA expression were observed at 12, 24 and 48 hrs, with an approximate two fold decrease in expression at 12 and 24 and a 1.5 fold increase in IE treatment group compared to nRBC controls at 48 hrs but the differences were not significant (all p values >0.05) (Figure 5).

**Figure 6.**
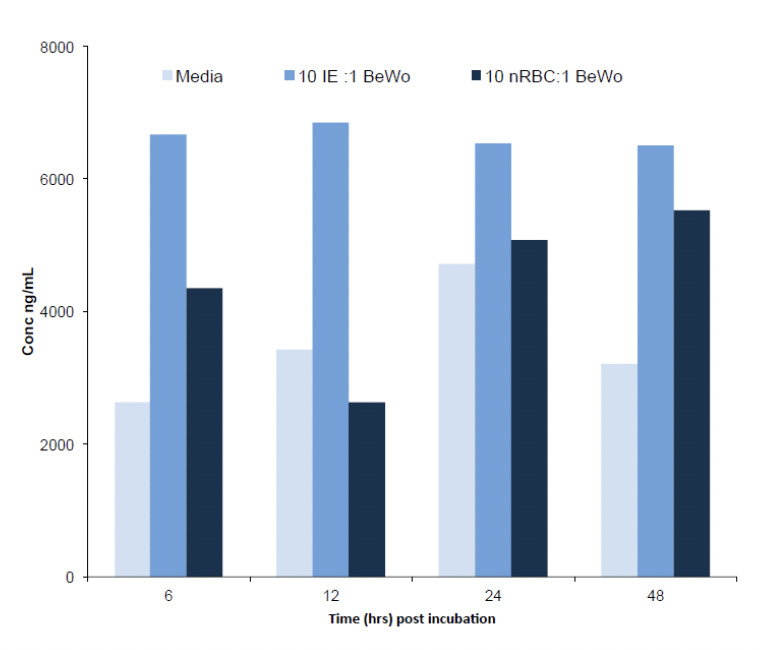
Time course expression of MIF (ng/ml) in BeWo cells. 2 x 10^5^ forskolin-treated BeWo were incubated with 2 x10^6^ CS2 IE using nRBC as control and incubated for 6, 12, 24 or 48 hrs. Supernatants were pooled from triplicates experiments and so only single assay of pooled samples were measured.

BeWo secreted MIF at all time points in all treatment, including the media control (Figure 6). However, a 2.5-fold increase in MIF concentration was found in the IE treatment group at 12 hrs compared to the amount induced by nRBC (Figure 6).

## 4 Discussion

In this study we sought to identify culture conditions for measuring parasite induced changes in BeWo cells with characteristics of ST. Incubation of BeWo with 10 μm of

forskolin at 72 hrs stimulated the cells to secrete higher levels of hCG compared at 24 and 48 hrs than untreated cells, indicating the cells had differentiated from cyto-trophoblasts into syncytial trophoblasts. Also to induce a significant response in BeWo cells, incubation with a ratio of intact 10 IE to BeWo was required, as no significant changes were seen when smaller numbers of IE were used. Most of the changes in RNA or protein expression between BeWo cultured with IE and nRBC were detected at 24 or 48 hrs post incubation, with differences being more apparent at 48 hrs. These results indicate that BeWo syncytial monolayers do not appear to respond to intact IE immediately after co-culture for the selected markers used in our study.

The results are also consistent with studies by Chaisavaneeyakorn *et al.* [21] who showed that IE induce significant MIF production in BeWo cells. Although the MIF assay in this study was descriptive, the pattern shows a 2-fold change in the level of MIF secreted by syncytialised BeWo cultured with 10 IE in the first 12 hrs after treatment. After 12 hrs, levels of MIF increased in both normal red blood cells and non treated BeWo; further validating earlier observations by Chaisavaneeyakorn *et al.* [15] that cytotophoblasts and ST intrinsically express MIF and that MIF is elevated in the intervillous space of women with placental malaria [13] may be secreted by other cells types in addition to ST.

Interleukin-8 is an important chemokine that recruits maternal monocytes to the intervillous space and is elevated there during placental malaria [22]. *In vivo* studies by Moormann *et al.* [23] indicated that maternal macrophages from placental malaria infected tissues express IL-8 and increased IL-8 RNA expression correlated with maternal monocytes in placental malaria infected placentas. We show a significant increase in IL-8 secretion in forskolin treated BeWo cultured at a 10 IE: 1 BeWo ratio after 48 hrs of incubation. Increase in

IL-8 secretion by BeWo treated with CSA-binding IE emphasises the immunological role of ST, as they recognise and respond to *P. falciparum* IE and its associated bioactive molecules. Induction of IL-8 secretion by BeWo in response to IE therefore demonstrates that ST are partly responsible to elevated levels of IL-8 *in vivo*.

*In vivo* studies have reported disregulation of growth factors in the intervillous space of placental malaria positive women [12,14,24-26]. Similarly, the current BeWo model (10 μm forskolin–treated BeWo for 72 hrs followed by incubation with 10 IE: 1 BeWo for 48 hrs) also results in significant changes in RNA expression of markers important for placental and fetal growth, including endoglin and VEGF A and marginally, but not statistically significant, RNA expression changes of mTOR, INHA and ANGPT-2 at 48 hrs. This is the first study to show that direct interaction of IE with ST actually lead to changes in expression of ST growth factors.

Recent studies of placental plasma from placental malaria positive women provided evidence for the disregula-tion of ST functions, including insulin like growth factor, leptin, VEGF, ANGPT-1/ ANGPT-2, soluble fms like tyrosine kinase 1 (sFLT), and soluble endoglin [12,14,24,25]. In these studies, important factors for fetus growth were dysregulated in placental malaria positive mothers who delivered low birth weight babies. To date, it is not fully understood if direct binding of IE or placental malaria associated inflammatory cytokines are responsible for the change in ST functions. It follows that if IE induces changes in ST that is associated with increased risk of fetal growth restriction, a vaccine that will generate antibodies to prevent binding of infected erythrocyte to ST and therefore sequestration, would be vital. However, if inflammation is responsible for the changes of ST function during placental malaria, then prompt and better diagnosis remains important. Therefore, this model can be used to help in understanding the pathophysiologic changes associated with placental malaria that leads to delivery of low birth weight babies.

Finally, our experimental study shows that 10 IE: induced expression of VEGF A and endoglin is significantly higher compared to the nRBC control indicating potential involvement of parasite derived ST responses during placental malaria. Increase in expression of an angiogenic molecule, i.e., VEGFA and endoglin in BeWo cells treated with IE, demonstrates the potential modulation of angiogenic processes by ST in response to placental malaria.

## 5 Conclusion

The results from this study have helped to identify culture conditions for use in studying malaria induced changes in BeWo as a model for events that occur in ST *in vivo* during placental malaria.
